# Ecotoxicological Assessment and Biodegradation of Prednisone by Aquatic Microorganisms

**DOI:** 10.3390/ijerph23040530

**Published:** 2026-04-18

**Authors:** Érika Michelle Miranda, Paula von Randow Cardoso, Carolina Paula de Souza Moreira, Marcos Paulo Gomes Mol

**Affiliations:** 1 Serviço de Ecotoxicologia, Diretoria de Pesquisa e Desenvolvimento, Fundação Ezequiel Dias, Belo Horizonte 30510-010, MG, Brazil; erika.miranda@funed.mg.gov.br (É.M.M.); paula.cardoso@funed.mg.gov.br (P.v.R.C.); 2 Serviço de Desenvolvimento Tecnológico Farmacêutico, Diretoria de Pesquisa e Desenvolvimento, Fundação Ezequiel Dias, Belo Horizonte 30510-010, MG, Brazil; carolina.moreira@funed.mg.gov.br

**Keywords:** ecotoxicity, biodegradation, prednisone, *Artemia salina*, *Aliivibrio fischeri*, *Microcystis novacekii*

## Abstract

**Highlights:**

**Public health relevance—How does this work relate to a public health issue?**

- The increasing release of pharmaceuticals into aquatic environments raises concerns about potential ecological and public health risks.
- This study investigates the ecotoxicity and biodegradation of prednisone, a widely used corticosteroid that may reach aquatic ecosystems through wastewater discharge.

**Public health significance—Why is this work of significance to public health?**

- Prednisone showed no detectable toxicity to representative aquatic organisms at concentrations up to its solubility limit (100 mg/L).
- The cyanobacterium *Microcystis novacekii* indirectly promoted prednisone degradation through pH changes in the culture medium.

**Public health implications—What are the key implications or messages for practitioners, policy makers and/or researchers in public health?**

- The findings contribute to environmental risk assessment of pharmaceuticals and support evidence-based monitoring of emerging contaminants in aquatic systems.
- Understanding environmental fate and degradation mechanisms of drugs can inform wastewater management strategies and environmental protection policies.

**Abstract:**

The increasing consumption of pharmaceuticals associated with global population growth has intensified concerns regarding their release into aquatic environments and potential ecotoxicological effects. In this context, this study evaluated the ecotoxicity and biodegradation of the widely used corticosteroid prednisone. Ecotoxicity assays were performed using aquatic organisms representing different trophic levels: *Artemia salina* (microcrustacean), *Aliivibrio fischeri* (marine bacterium), and the cyanobacterium *Microcystis novacekii*. Biodegradation assays were conducted using *M. novacekii*. Prednisone was tested at concentrations ranging from 5 to 100 mg/L, corresponding to its maximum solubility in water. All experiments were carried out in accordance with standardized protocols (ABNT NBR 16530, ABNT NBR 15411-3, ISO 11348-3, and OECD 201). No toxic effects were observed for prednisone in any of the tested organisms, as responses at all tested concentrations, including the highest, were not significantly different from the control. Therefore, it was not possible to estimate EC50 values within the tested concentration range. According to the Globally Harmonized System of Classification and Labelling of Chemicals (GHS), substances with effect concentrations above 100 mg/L are considered non-toxic to aquatic organisms. During biodegradation assays, a reduction in prednisone concentration was observed during the growth of *M. novacekii*, which was associated with an increase in the pH of the culture medium. These results suggest that prednisone degradation occurred indirectly through pH changes caused by cyanobacterial growth rather than through direct metabolic pathways.

## 1. Introduction

Global concern regarding environmental degradation has intensified in recent decades. Population growth and the rising prevalence of acute and chronic illnesses have driven a substantial increase in the consumption of pharmaceuticals and personal care products for both human and veterinary applications. The residues of these compounds are frequently released into the environment without adequate treatment, posing a growing risk of contamination. Numerous studies have detected pharmaceuticals in surface and groundwater worldwide, typically at concentrations ranging from ng/L to µg/L, and in some cases reaching mg/L levels [1,2,3,4,5]. The detection of these substances, classified as anthropogenic or naturally occurring contaminants, has heightened global concern regarding their environmental persistence and potential ecological impacts [6].

Pharmaceuticals may enter aquatic environments through treated or untreated wastewater originating from households, hospitals, pharmaceutical industries, livestock production, and landfill leachates. Several of these compounds are regarded as pseudo-persistent due to their continuous release and sustained environmental input. A prominent example is the spread of antimicrobial resistance, a major global public health threat, which may be exacerbated by the misuse of antibiotics and their inadequate disposal, resulting in environmental contamination. In aquatic ecosystems, antibiotics can affect not only target pathogenic microorganisms but also non-target microbial communities due to their broad-spectrum activity and limited biological specificity, leading to disruptions in microbial structure and function, including alterations in nutrient cycling and ecosystem processes [7,8].

Despite the availability of various wastewater treatment technologies, conventional systems are often ineffective at removing or degrading toxic and persistent compounds [9,10]. According to Tran, Reinhard, and Gin (2018), municipal wastewater treatment plants are primarily designed to reduce carbonaceous, nitrogenous, and phosphorous loads rather than eliminate micropollutants, particularly those that are recalcitrant or hazardous [11]. These systems typically encompass primary, secondary, and occasionally tertiary treatment processes, which are insufficient for the complete removal of pharmaceutical contaminants.

Prednisone is a synthetic glucocorticoid belonging to the class of systemic corticosteroids, widely prescribed for its anti-inflammatory and immunosuppressive properties. It is indicated for a broad range of disorders, including endocrine, musculoskeletal, rheumatic, and neoplastic diseases, among others [12]. Once administered, prednisone acts as a prodrug and is primarily converted in the liver to its pharmacologically active form, prednisolone, by the enzyme 11β-hydroxysteroid dehydrogenase. This interconversion is reversible and may also occur in extrahepatic tissues such as adipose tissue, bone, skin, and ocular tissues. However, the extent of systemic availability and subsequent excretion of prednisolone may differ depending on whether it is formed in vivo from prednisone or directly administered, due to factors such as first-pass metabolism, tissue distribution, plasma protein binding, and differences in metabolic pathways. In both cases, only the unbound fraction of prednisolone is biologically active and able to diffuse across cellular membranes [13,14].

Glucocorticoids are lipophilic and bind to receptors expressed in nearly all tissues [13]. These compounds may act through membrane-associated receptors or translocate to the nucleus to modulate gene expression by activating or repressing specific signaling pathways [15]. Regarding the pharmacokinetics of prednisone, approximately 3 ± 2% of the administered dose is excreted unchanged in urine, with an additional 15 ± 5% excreted as prednisolone. Conversely, prednisolone is excreted unchanged at levels of approximately 26 ± 9%, with an additional 3 ± 2% excreted as prednisone [14].

Given the environmental challenges posed by pharmaceutical contamination, this study evaluated the aquatic toxicity of prednisone using biological models that remain underexplored for this compound. Additionally, the study examined the biodegradation potential of prednisone by a cyanobacterial species. A literature review showed an absence of ecotoxicity data for prednisone in most of the organisms employed here, as well as a lack of studies assessing its biodegradation by cyanobacteria. Thus, this study aimed to assess the acute toxicity of the active pharmaceutical ingredient (API) prednisone in the microcrustacean *Artemia salina* and the marine bacterium *Aliivibrio fischeri*, to evaluate both acute and chronic toxicity in the cyanobacterium *Microcystis novacekii*, and to investigate the biodegradation of prednisone by *M. novacekii* through quantification of the compound in the culture medium.

## 2. Materials and Methods

### 2.1. Preliminary Procedures

Initial procedures included evaluating the solubility of prednisone (PRED) in water, as aqueous media serve as the basis for all bioassays and for determining the concentration range for subsequent testing. A standard calibration curve was also prepared using a certified secondary reference standard to quantify PRED in both the raw material and experimental samples. In addition, the growth curve of the cyanobacterium *M. novacekii* was established to support acute and chronic toxicity assays and the biodegradation experiments. Acute toxicity tests were also conducted for *A. salina* and *A. fischeri*.

#### 2.1.1. Prednisone Solubility Assessment

According to the Brazilian Pharmacopoeia, prednisone is classified as “sparingly soluble” in water [16]. PubChem reports a solubility of 77.54 mg/L at 25 °C. Therefore, the first ecotoxicity assay, conducted with *A. salina*, employed a maximum test concentration of 75 mg/L. Subsequently, solubility was reassessed using mild heating (up to 55 °C) under agitation, based on the compound’s thermal stability [17,18,19]. This procedure allowed the preparation of solutions up to 100 mg/L without precipitation after cooling. Thus, assays with *A. fischeri* and *M. novacekii* were conducted using concentrations up to 100 mg/L.

#### 2.1.2. Preparation of Calibration Curves and Chromatographic Analysis

A pharmaceutical-grade secondary standard of prednisone (Sigma Aldrich, St. Louis, MO, USA, PHR 1042-1G, source LRA 02231) was used to prepare calibration curves in all media employed in the assays (ASM-1, saline solution 3.5%, and 2.0% NaCl), as well as in ultrapure water. Steril ASM-1 medium was used for the cyanobacterium, 3.5% saline solution was used for *A. salina*, and 2.0% NaCl solution was used for *A. fischeri*, according to standard cultivation and testing protocols. Stock solutions of 100 mg/L were prepared in triplicate with heating, and serial dilutions were made to obtain seven calibration points: 100, 50, 25, 12.5, 6.25, 3.125, and 1.5625 mg/L.

Ultrapure water was used exclusively for the preparation of all media and stock solutions, ensuring the absence of contaminants that could interfere with both biological assays and chromatographic analysis. The dilution of the PRED stock solution was performed using the respective test media for each organism, not ultrapure water, in order to maintain consistent exposure conditions.

Chromatographic analyses were performed using a Shimadzu LC-2060C 3D system (Shimadzu Corporation, Kyoto, Japan) equipped with a Discovery^®^ HS C18 column (25 cm × 4.6 mm, 5 μm) (Avantor, Radnor, PA, USA). The mobile phase consisted of a methanol (B) and water (A) gradient: 65% B for 5 min, 65–85% B from 5–6 min, 85% B from 6–15 min, followed by re-equilibration, totaling a 20-min run. The flow rate was 0.5 mL/min, the injection volume was 10 μL, and the detection wavelength was 254 nm. Retention time (RT) was approximately 9.3 min. Data acquisition and processing were performed in LabSolutions (version 5.136).

Calibration curves were constructed by plotting concentration versus chromatographic peak area, and sample quantification was conducted via linear regression. The prednisone (PRED) raw material used in the experiments (manufacturer: Hunan Yunxin (Hunan Yuxin Pharmaceutical Co., Ltd., Changsha, China); batch PLS191001) was analyzed by preparing 50 mg/L solutions in triplicate.

### 2.2. Ecotoxicity Assays

#### 2.2.1. Cyanobacteria (*M. novacekii*) Toxicity Assay

Growth curves were established for *M. novacekii* (strain BA005) using cell counts in a Fuchs Rosenthal chamber and absorbance measurements at 680 nm (GE Ultrospec 2100 (GE Healthcare, Boston, MA, USA)). The strain, isolated in 2004 from Lake Dom Helvécio (Rio Doce State Park, Marliéria, Brazil), was obtained from the algal culture bank of the LIMNEA Laboratory (UFMG) [20]. Methodological procedures followed OECD Guideline 201 and established cyanobacterial cultivation protocols [21,22,23].

Cultures were maintained at 23 ± 1 °C under a 10 h light/10 h dark photoperiod (1600 lux). Growth was monitored for 21 days with measurements on days 5, 9, 13, 17, and 21, yielding a growth regression of R^2^ = 0.992 (y = 20,000,000x + 352,778). Monthly transfers during the logarithmic phase ensured culture viability.

Acute toxicity assays (96 h) and one chronic/biodegradation assay (21 days) were conducted using an initial inoculum of 1 × 10^5^ cells/mL [21]. Copper sulfate (2 mg/L) served as the positive control. Five concentrations of prednisone (100, 50, 25, 12.5, and 6.25 mg/L) were prepared in ASM-1 medium, each in triplicate (50 mL per experimental unit). Growth controls and blanks (media containing each concentration of PRED without inoculum) were included to monitor chemical stability.

Measurements of pH and optical density (OD680) began 18–24 h after assay initiation and continued daily during acute tests and on specified days for chronic assays. Cell counts were calculated from OD values using the growth regression model, and growth rates were subsequently determined. For the analytical measurements, independent aliquots were pipetted for each parameter.

#### 2.2.2. *Artemia salina* Acute Toxicity Assay

Assays with *A. salina* were performed according to ABNT NBR 16530 [24]. Organisms were cultured in 3.5% saline solution (commercial marine salt commonly employed in aquaculture), pH 8.5. Sodium dodecyl sulfate (0.2%) was used as a positive control. Cysts were incubated at 27–28 °C, and nauplii were transferred to fresh saline after 24 h. Metanauplii (48 h) were exposed for 24 h to five PRED concentrations (75, 50, 25, 10, and 5 mg/L). Ten organisms were added per tube, and the assay was performed in quadruplicate across three independent assays. Mobility was assessed visually as the endpoint.

#### 2.2.3. *Aliivibrio fischeri* Luminescence Inhibition Assay

Luminescence inhibition assays followed ABNT NBR 15411-3, ISO 11348-3, and CETESB technical standard L5.227 [25,26,27]. Lyophilized *A. fischeri* (Biolux^®^ Lyo kit (Bioscience International, Inc., Rockville, MD, USA)) was reconstituted and analyzed using a BioFix^®^ Lumi-10 luminometer (BioTek Instruments, Inc., Winooski, VT, USA).

Prednisone serial dilutions were prepared in 2% NaCl. Stock solution (100 mg/L) was prepared, aerated for ~10 min, and serially diluted to obtain 50, 25, 12.5, and 6.25 mg/L. Initial luminescence was recorded immediately after bacterial addition (time zero). Prednisone solutions at the specified concentrations were added to the corresponding tubes containing the luminescent bacterial inoculum. Luminescence was then measured at 15 and 30 min. The Biolux assay followed a sequential dilution procedure, in which solutions prepared in series A were transferred to series B cuvettes containing 0.1 mL of bacterial inoculum, resulting in final PRED concentrations of 90, 45, 22.5, 11.25, and 5.625 mg/L. The pH of all solutions was adjusted to 6.0–8.5.

Positive controls consisted of potassium dichromate (100 mg/L). All assays were performed in triplicate in five independent experiments, exceeding the minimum rigor of the referenced standards.

### 2.3. M. novacekii Biodegradation Assays

Biodegradation assays with *M. novacekii* followed the same procedures described previously as the ecotoxicity tests, but were extended to 31 days. Prednisone was tested at 100, 50, and 6.25 mg/L in triplicate, along with growth controls, positive controls, and blanks (PRED in ASM-1 without inoculum). Twice-weekly aliquots were filtered (0.22 µm, Merck Millipore, Darmstadt, Germany) and analyzed via HPLC to determine PRED concentration. Degradation rates (percentage reduction per day) were calculated for each concentration and compared to blanks. Chronic toxicity to cyanobacteria was also assessed during these assays.

### 2.4. Statistical Analysis

The statistical analysis initially followed the recommendations of the Organisation for Economic Co-operation and Development Test Guideline 201, in which concentration response relationships are modeled using nonlinear regression (e.g., logistic, Weibull, and log-normal models) to estimate ECx values with 95% confidence intervals. However, none of these models provided an adequate fit to the data due to the absence of a clear monotonic concentration–response pattern. Alternative models for non-monotonic or hormetic responses were considered but not applied due to insufficient evidence of consistent hormesis and the risk of overfitting. Therefore, a hypothesis-testing approach was adopted, using one-way ANOVA followed by Dunnett’s test to compare treatments with the control and derive NOEC/LOEC values. No statistically significant differences were observed, indicating that the tested substance did not affect the growth rate under the experimental conditions.

Statistical analyses were performed using RStudio 2025.05.0. Data normality was evaluated using the Shapiro–Wilk test (α = 0.05). Parametric comparisons were conducted using Student’s *t*-test, whereas nonparametric comparisons employed the Mann–Whitney test, both with 95% confidence intervals. ANOVA was applied to evaluate differences in calibration curve slopes among the various culture media and water, assessing potential matrix effects.

## 3. Results and Discussion

Standard solutions of PRED were analyzed by HPLC to construct calibration curves used for quantification in all assays. Statistical evaluation of curve slopes using ANOVA revealed no significant matrix effect among the calibration curves obtained in the different media (*p* = 0.199). The prednisone raw material used in the study showed an assay value of 102.18%, confirming the accuracy of theoretical concentrations applied in the experiments.

### 3.1. Ecotoxicity Assays

**Figure 1:**
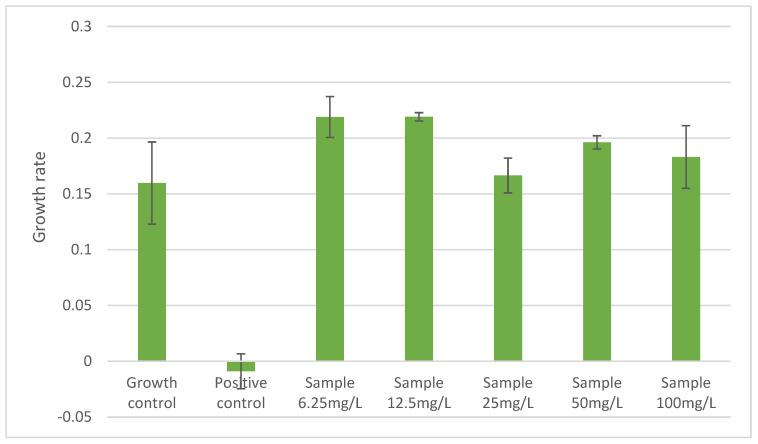
Effect of prednisone concentrations on the growth rate of the cyanobacterium *M. novacekii* after 96 h of exposure. Acute toxicity assays (96 h) were performed and statistical comparisons between each tested concentration and the control at 96 h showed *p* > 0.05, indicating no significant differences.

Acute and chronic toxicity tests revealed no inhibitory effects of PRED on the growth of *M. novacekii*. Cyanobacterial growth was sustained in all tested concentrations and was comparable to growth controls (Figure 1). Statistical comparisons between treatments and controls consistently showed no significant differences, supporting the absence of toxic effects.

**Figure 2:**
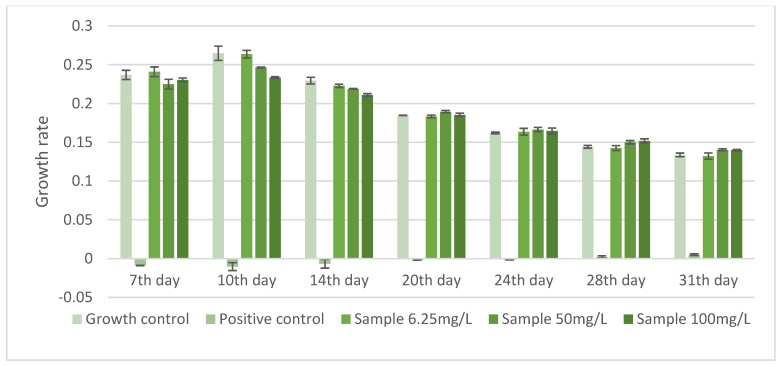
Growth rate of the cyanobacterium *M. novacekii* exposed to different concentrations of prednisone during 31-day assays. Chronic toxicity tests (31 days) were conducted and statistical comparisons between each concentration and the control were performed for each reading day in each assay with no significant differences observed (*p* > 0.05).

The chronic exposure assays showed no statistically significant differences relative to the growth control (Figure 2). Consistent with the acute toxicity results, the responses were stable across treatments and experimental days, with no evidence of a concentration-response pattern, indicating the absence of detectable effects of prednisone under the evaluated conditions.

**Figure 3:**
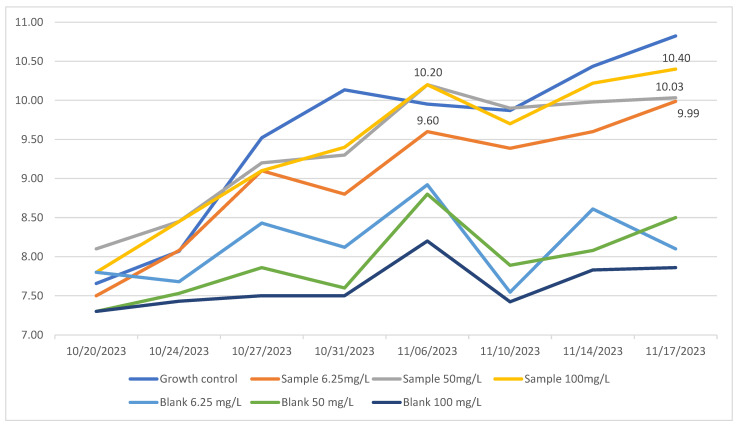
Variation of pH during the 31-day assays of the cyanobacterium *M. novacekii* exposed to different concentrations of prednisone, during chronic toxicity tests (31 days).

Consistent increases in pH were observed across all treatments and controls during the chronic exposure tests (Figure 3). This increase is characteristic of normal cyanobacterial autotrophic metabolism, which consumes inorganic carbon and releases hydroxyl ions, thereby raising the pH [28]. These findings further confirm the lack of inhibitory effects of PRED on cyanobacterial viability.

**Figure 4:**
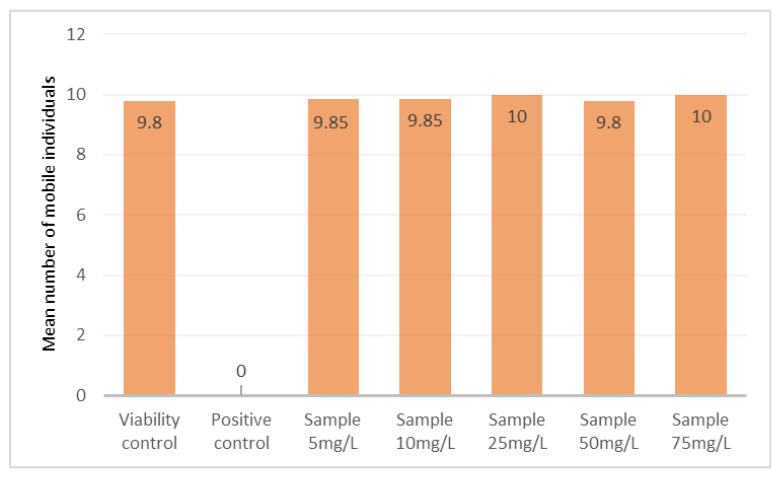
Mean number of mobile individuals of *A. salina* after exposure to prednisone (sample). Statistical comparisons between the tested concentrations and the viability control showed *p* > 0.05, indicating no significant differences.

The acute toxicity assay with *A. salina* showed no reduction in organism mobility across all tested concentrations of PRED (Figure 4). Metanauplii exhibited approximately 100% mobility, similar to the viability controls. Statistical comparison between treatments and controls revealed no significant differences (*p* > 0.05), indicating that soluble PRED, even at its maximum aqueous solubility (~75 mg/L), did not produce toxic effects in this organism.

**Figure 5:**
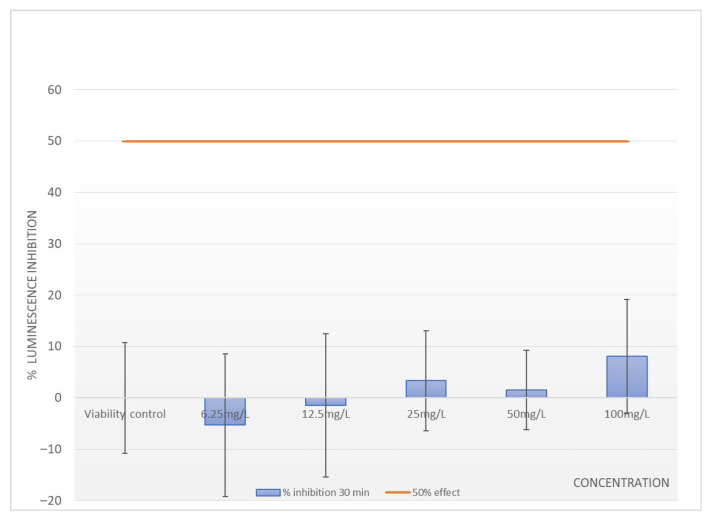
Percentage of luminescence inhibition of *A. fischeri* after 30 min exposure to prednisone across all tests.

Luminescence inhibition assays demonstrated no significant toxic effects of PRED on *A. fischeri*. At the highest test concentration (90 mg/L), average luminescence inhibition after 30 min was approximately 8% (Figure 5). However, statistical analyses showed no significant differences between treatments and controls (*p* > 0.05), confirming the absence of acute toxicity.

Under the Globally Harmonized System (GHS), substances with EC50 values greater than 100 mg/L are classified as non-toxic to aquatic organisms. Because no measurable effects were observed across the tested concentration range, the dataset did not include the partial or high inhibition levels required to establish a reliable concentration-response relationship. Consequently, a valid dose-response model could not be fitted and an EC50 value could not be calculated. These findings indicate that the EC50 is likely higher than the maximum tested concentration (100 mg/L), suggesting that PRED does not meet the criteria for acute or chronic aquatic toxicity classification under the GHS.

Although prednisone itself exhibited no toxicity in the present study, its active metabolite prednisolone has shown toxicity in aquatic vertebrates and invertebrates, including *Ceriodaphnia dubia*, *Danio rerio*, and *Physa acuta* [29,30,31]. Given that prednisone is partially excreted as prednisolone [14] and that conversion of prednisone to prednisolone is favored over the reverse reaction [32], potential environmental risks may arise indirectly through metabolite formation.

A comparative compilation of ecotoxicity data from the literature also supports the findings presented here, with previous studies reporting negligible toxicity of PRED. Differences observed in vertebrate species may be attributed to the presence of glucocorticoid receptors, endocrine signaling pathways, and the enzyme 11β-hydroxysteroid dehydrogenase, which enables conversion of prednisone to its active form [33,34].

The absence of toxicity in *M. novacekii* is consistent with the known resilience of cyanobacteria to xenobiotics, attributed to their physiological adaptability, mucilaginous extracellular layer, and production of extracellular polymeric substances (EPS) capable of providing physical and chemical protection [23,35,36]. Cyanobacteria are therefore widely used in bioremediation processes and biodegradation assessments [37,38,39].

Environmental detections of PRED typically occur at nanogram-to-microgram levels, as reported in Spain (21–285 ng/L), the Netherlands (117–545 ng/L), the United Kingdom (30–850 ng/L), and Brazil (up to 8105 ng/L) [2,3,4,5,40,41]. Although environmental levels are much lower than those tested here, experimental exposures at solubility limits reflect a worst-case scenario and offer insight into potential hazards associated with accidental releases or industrial effluents.

Despite the findings, uncertainties remain regarding long-term effects and impacts on species not evaluated in this study. As emphasized by Bal and coworkers and Cole & Brooks, the environmental behavior and ecotoxicity of glucocorticoids are still poorly understood [29,42].

### 3.2. Biodegradation Assays

**Figure 6:**
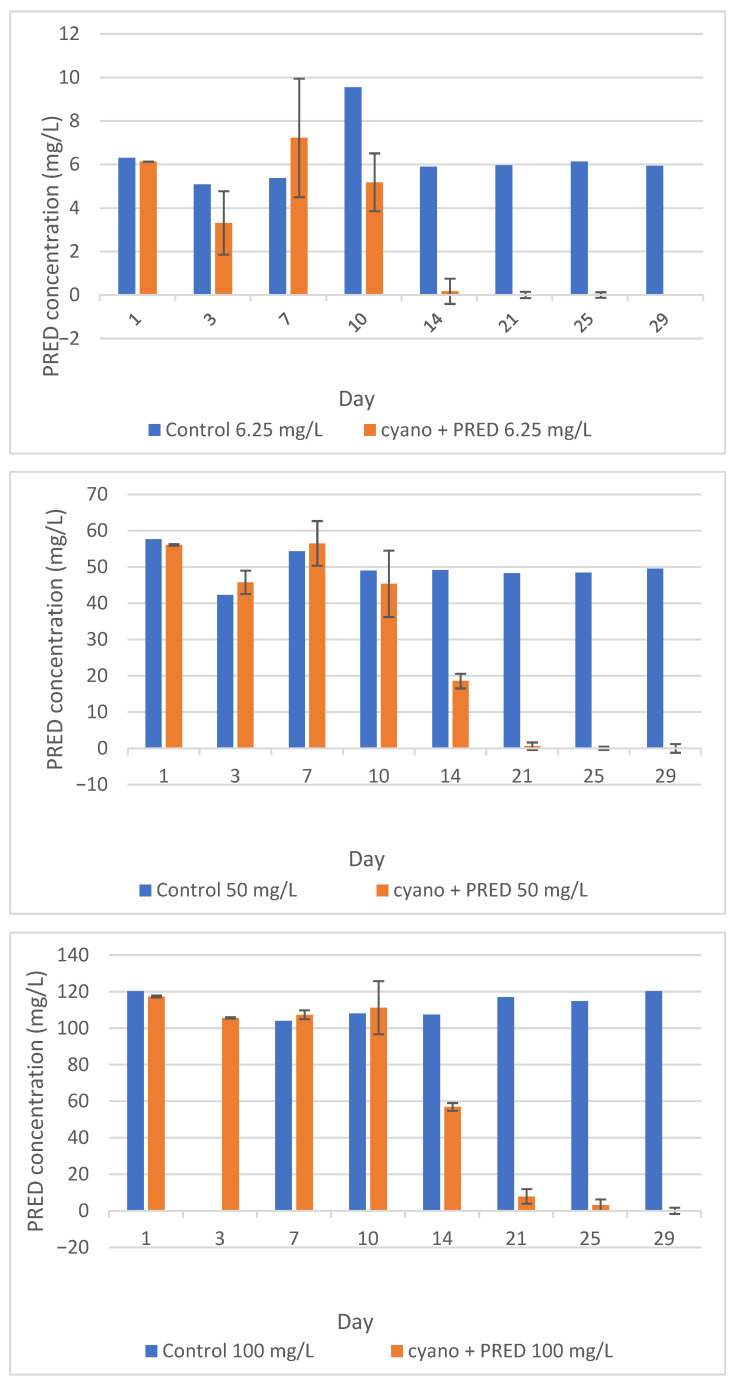
Evaluation of PRED degradation by the cyanobacterium *M. novacekii* over the experimental period, for different initial concentrations of PRED.

Biodegradation assays conducted over 31 days revealed distinct patterns among the three experimental concentrations (6.25, 50, and 100 mg/L). Figure 6 illustrates that PRED concentrations in blank samples remained stable, confirming chemical integrity under the conditions tested. In contrast, samples inoculated with *M. novacekii* showed significant reductions in PRED (Figure 6).

Growth rate and pH data (Figure 2 and Figure 3) revealed a strong relationship between cyanobacterial metabolic activity and PRED concentration reduction. It can be noted that cyanobacterial growth peaked was accompanied by substantial increases in pH (up to 10), coinciding with the most pronounced decreases in PRED concentration. In contrast, lower growth rates and more moderate pH increases (maximum ~9) were associated with limited PRED concentration reduction.

**Figure 7:**
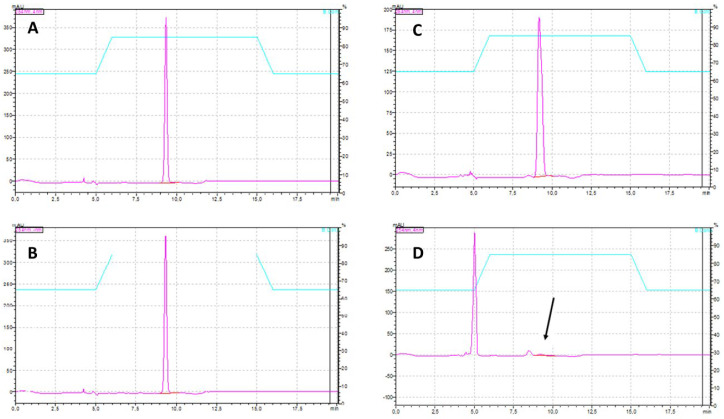
Chromatographic comparison of prednisone (PRED) in control (blank) samples and in assays containing the cyanobacterium *M. novacekii* at the beginning and after 28 days of the 4th experiment. (**A**) PRED peak in the blank at day 0; (**B**) PRED peak in the blank at day 28; (**C**) PRED peak in the 100 mg/L sample at day 0; (**D**) absence of the PRED peak in the 100 mg/L sample after 28 days.

Chromatograms (Figure 7) show disappearance of the PRED peak in inoculated samples, with appearance of a degradation product at ~5 min retention time. Although not the focus of this study, this observation suggests degradation under alkaline conditions. Three potential mechanisms were evaluated to explain the PRED degradation: biodegradation by cyanobacteria; abiotic degradation driven by increased pH; and adsorption to cyanobacterial mucilage (secondary mechanism).

Although *M. novacekii* produces enzymes such as 7α-hydroxysteroid dehydrogenase, which could theoretically convert PRED to prednisolone [43], the metabolic and physicochemical profiles observed in this study do not support biodegradation as the primary mechanism. If PRED were being metabolized as a carbon source, a shift toward heterotrophic metabolism would be expected, typically resulting in medium acidification due to CO_2_ production [28]. In contrast, a consistent increase in pH was observed across all assays, indicating that cyanobacterial metabolism remained predominantly autotrophic. This alkalinization, driven by photosynthetic CO_2_ uptake, provides strong evidence against the microbial utilization of PRED as a substrate.

The increase in pH is directly linked to the observed reduction in PRED concentration. Under alkaline conditions, particularly above pH 10, corticosteroids such as prednisone are susceptible to abiotic transformation via base-catalyzed hydrolysis and associated structural rearrangements (e.g., keto–enol tautomerism and cleavage of functional groups within the steroid nucleus) [13,43,44,45]. These reactions lead to chemical degradation independent of biological activity. In this context, the temporal correspondence between rising pH and decreasing PRED concentration strongly supports a mechanism of pH-driven abiotic degradation.

Furthermore, the pattern of PRED removal does not correlate with biomass accumulation, which would be expected if biodegradation were a dominant process. Specifically, no enhanced removal was observed during periods of maximum biomass, and in some assays (e.g., the 5th), substantial biomass was present with only minimal PRED reduction. These observations further weaken the hypothesis of direct metabolic degradation.

Adsorption to extracellular mucilage cannot be fully excluded; however, several observations argue against this being the dominant mechanism: minimal reduction in the 5th assay despite substantial biomass; lack of early-phase PRED removal during peak growth (when adsorption would be strongest); and absence of retention during filtration prior to HPLC analysis. Thus, adsorption may contribute marginally, but it does not explain the pattern observed across assays.

Taken together, the results indicate that *M. novacekii* does not directly biodegrade prednisone under the tested conditions. Instead, cyanobacterial activity indirectly promotes PRED removal by increasing the pH of the medium through autotrophic metabolism, thereby creating conditions that favor abiotic degradation. This distinction between direct biodegradation and environmentally mediated transformation is essential for correctly interpreting the fate of PRED in cyanobacteria-containing systems.

## 4. Conclusions

This study evaluated the ecotoxicity and environmental behavior of the pharmaceutical prednisone, a compound of environmental concern due to its widespread therapeutic use and frequent detection in aquatic systems. The results demonstrated that prednisone did not exhibit acute or chronic toxicity to the tested aquatic organisms, as no adverse effects were observed at concentrations up to its solubility limit (100 mg/L) for *A. salina, A. fischeri,* and *M. novacekii*. According to the GHS criteria [46], substances with effect concentrations above 100 mg/L are considered non-toxic to aquatic organisms, indicating that prednisone presents low ecotoxicological risk under the evaluated conditions.

The ecotoxicological assessment was conducted using test systems aligned with internationally recognized approaches, including the Microtox assay and growth inhibition assays, which are conceptually consistent with OECD/ISO standards for microbial toxicity and primary producer testing. The absence of measurable effects across these systems suggests a low potential for acute toxicity. However, according to recent advances in OECD/ISO-based frameworks for microbial toxicity assessment, a more comprehensive evaluation would benefit from the inclusion of additional endpoints, particularly those related to microbial community function and wastewater treatment processes [47].

From a biodegradation perspective, standardized OECD/ISO test systems represent the benchmark for evaluating ready and inherent biodegradability [48]. In this context, prednisone appears to be a challenging compound to assess, as the results of the present study indicate that its removal is predominantly governed by abiotic transformation processes rather than true microbial mineralization. Specifically, the reduction in prednisone concentration observed during the cyanobacterial assays was associated with an increase in pH above 10, driven by autotrophic activity, which favored chemical degradation via alkaline hydrolysis. This indicates that environmental dissipation may occur indirectly, without significant biological degradation.

Overall, the results suggest that prednisone poses a low ecotoxicological hazard to aquatic organisms under the tested conditions. Nevertheless, future studies should incorporate additional OECD/ISO-based microbial toxicity assays and standardized biodegradation tests, as well as focus on identifying transformation products formed under alkaline conditions and evaluating their persistence and potential ecological effects.

## Data Availability

The original contributions presented in this study are included in the article.

## Abbreviations

The following abbreviations are used in this manuscript:

ABNT	Associação Brasileira de Normas Técnicas
ANOVA	Analysis of Variance (Análise de Variância)
API	Active Pharmaceutical Ingredient (Ingrediente Farmacêutico Ativo)
ASM-1	Algal Synthetic Medium-1
CC BY	Creative Commons Attribution License
CETESB	Companhia Ambiental do Estado de São Paulo
EC50	Median Effect Concentration
EPS	Extracellular Polymeric Substances
GE	General Electric
GHS	Globally Harmonized System of Classification and Labelling of Chemicals
HPLC	High-Performance Liquid Chromatography
ISO	International Organization for Standardization
NaCl	Sodium Chloride
OD680	Optical Density at 680 nm
OECD	Organisation for Economic Co-operation and Development
PRED	Prednisone
RT	Retention Time
UFMG	Universidade Federal de Minas Gerais

## References

[1] Xiang Y., Wu H., Li L., Ren M., Qie H., Lin A. (2021). Distribution and risk of PPCPs in China’s aquatic environment. Ecotoxicol. Environ. Saf..

[2] Schriks M., van Leerdam J.A., van der Linden S.C., van der Burg B., van Wezel A.P., de Voogt P. (2010). High-Resolution Mass Spectrometric Identification and Quantification of Glucocorticoid Compounds in Various Wastewaters in The Netherlands. Environ. Sci. Technol..

[3] Kugathas S., Williams R.J., Sumpter J.P. (2012). Predicted environmental concentrations of glucocorticoids in the River Thames. Environ. Int..

[4] Santos A.V., Couto C.F., Lebron Y.A.R., Moreira V.R., Foureaux A.F.S., Reis E.O., Santos L.V.d.S., de Andrade L.H., Amaral M.C.S., Lange L.C. (2020). Occurrence and risk assessment of pharmaceuticals in Brazilian water supply systems. Sci. Total Environ..

[5] Reis E.O., Foureaux A.F.S., Rodrigues J.S., Moreira V.R., Lebron Y.A.R., Santos L.V.S., Amaral M.C.S., Lange L.C. (2019). Occurrence and removal of pharmaceuticals in Brazilian drinking water treatment plants. Environ. Pollut..

[6] Kumar R., Qureshi M., Vishwakarma D.K., Al-Ansari N., Kuriqi A., Elbeltagi A., Saraswat A. (2022). Emerging water contaminants and sustainable removal technologies. Case Stud. Chem. Environ. Eng..

[7] Xie H., Hao H., Xu N., Liang X., Gao D., Xu Y., Gao Y., Tao H., Wong M. (2019). Pharmaceuticals and personal care products in water, sediments, aquatic organisms, and fish feeds in the Pearl River Delta: Occurrence, distribution, potential sources, and health risk assessment. Sci. Total Environ..

[8] Lima D.R.S., Tonucci M.C., Libânio M., de Aquino S.F. (2017). Pharmaceuticals and endocrine disruptors in Brazilian waters. Eng. Sanit. Ambient..

[9] Laitano K.S., Matias W.G. (2006). Toxicity tests with Daphnia magna: A tool for evaluation of an experimental UASB reactor. J. Braz. Soc. Ecotoxicol..

[10] Arpin-Pont L., Bueno M.J.M., Gomez E., Fenet H. (2016). Occurrence of PPCPs in the marine environment: A review. Environ. Sci. Pollut. Res..

[11] Tran N.H., Reinhard M., Gin K.Y. (2018). Occurrence and fate of emerging contaminants in municipal wastewater treatment plants from different geographical regions-a review. Water Res..

[12] Brasil, Fundação Oswaldo Cruz (2006). Therapeutic Handbook—Prednisone.

[13] Toledo L. (2020). Development and Validation of a Stability-Indicating Method for Prednisone Tablets. Master’s Thesis.

[14] Brunton L.L., Chabner B.A., Knollmann B.C. (2012). Goodman & Gilman’s the Pharmacological Basis of Therapeutics.

[15] Castro P.H.C. (2022). Action of Prednisone and Prednisolone on Kv1.3 Potassium Channel. Master’s Thesis.

[16] ANVISA (2019). Brazilian Pharmacopoeia.

[17] FUNED (2020). Prednisone Raw Material Study and Pre-Formulation Report.

[18] DrugBank Prednisone. https://www.drugbank.ca/drugs/DB00635.

[19] PubChem Prednisone. https://pubchem.ncbi.nlm.nih.gov/compound/5865.

[20] Barbosa F.A.R., Carvalho P.I.A. (2017). Algae and Microalgae Culture Collection—LIMNEA Laboratory.

[21] OECD (2011). Test No. 201: Freshwater Alga and Cyanobacteria, Growth Inhibition Test.

[22] Jacinavicius F.R., Junior W.A.G., Azevedo M.T.d.P., Sant C.L. (2013). Manual for Cyanobacteria Cultivation.

[23] Wojciechowski J. (2013). Isolation and Cultivation of Microalgae.

[24] (2021). Aquatic Ecotoxicology—Acute Toxicity Test with Artemia sp. (Crustacea, Branchiopoda).

[25] (2021). Aquatic Ecotoxicology—Inhibitory Effect on the Bioluminescence of Vibrio fischeri. Part 3: Method Using Lyophilized Bacteria.

[26] (2008). Water Quality—Determination of the Inhibitory Effect of Water Samples on the Light Emission of Vibrio fischeri.

[27] CETESB (2001). Toxicity Test with Luminescent Bacteria Vibrio fischeri.

[28] Kim S., Park J.-E., Cho Y.-B., Hwang S.-J. (2013). Growth and nutrient removal by *Chlorella sorokiniana* under different trophic modes. Bioresour. Technol..

[29] Bal N., Kumar A., Du J., Nugegoda D. (2017). Multigenerational effects of two glucocorticoids (prednisolone and dexamethasone) on life-history parameters of crustacean Ceriodaphnia dubia (Cladocera). Environ. Pollut..

[30] Chen Q., Li C., Gong Z., Chan E.C.Y., Snyder S.A., Lam S.H. (2017). Common deregulated gene expression profiles and morphological changes in developing zebrafish larvae exposed to environmental-relevant high to low concentrations of glucocorticoids. Chemosphere.

[31] Bal N., Kumar A., Nugegoda D. (2017). Assessing multigenerational effects of prednisolone to the freshwater snail, Physa acuta (Gastropoda: Physidae). J. Hazard. Mater..

[32] Kyler K.E., Szefler S.J. (2024). Fifty years of unraveling the clinical pharmacology of corticosteroids. J. Pharm. Sci..

[33] Souza K.C. (2019). Influence of Water Temperature on Reproductive Performance of Betta splendens. Master’s Thesis.

[34] Thibault O., Cubbage T., Brink M., McCarthy J., Gunn C., Torres I., Faulkner P.C., Hala D., Petersen L.H. (2021). The pharmaceutical prednisone affects sheepshead minnow (*Cyprinodon variegatus*) metabolism and swimming performance. Comp. Biochem. Physiol. A.

[35] Silva S.R. (2017). Toxicity and Degradation of Antiretroviral Tenofovir Disoproxil Fumarate. Ph.D. Thesis.

[36] Araújo M. Cyanobacteria. https://www.infoescola.com.

[37] Melo I.S. Bioremediation. https://www.embrapa.br.

[38] Wang Q., Liu W., Li X., Wang R., Zhai J. (2020). Carbamazepine toxicity and its co-metabolic removal by the cyanobacteria *Spirulina platensis*. Sci. Total Environ..

[39] Guo Z., He H., Yang G., Liu K., Xi Y., Li Z., Luo Y., Liao Z., Dao G., Ren X. (2024). Environmental risks of antiviral drug arbidol in eutrophic lakes: Interactions with *Microcystis aeruginosa*. J. Hazard. Mater..

[40] Herrero P., Borrull F., Pocurull E., Marcé R. (2012). Determination of glucocorticoids in sewage and river waters by ultra-high performance liquid chromatography-tandem mass spectrometry. J. Chromatogr. A.

[41] Cole A.R., Brooks B.W. (2023). Global occurrence of synthetic glucocorticoids and glucocorticoid receptor agonistic activity, and aquatic hazards in effluent discharges and freshwater systems. Environ. Pollut..

[42] UniProt 7-Alpha-Hydroxysteroid Dehydrogenase (A0A552J6X9). https://www.uniprot.org/uniprotkb/A0A552J6X9.

[43] Müller C., Godoy G., Diego M. (2011). Chemical stability of prednisone oral suspension. J. Chil. Chem. Soc..

[44] Amaral M.C.S., Ricci B., Madeira A., Marinho B.M.A., Figueiró A.M.J. (2019). Evaluation of the biodegradability and inhibitory effects associated with anaerobic digestion of pharmaceuticals. Proceedings of the 30th ABES Congress—Brazilian Congress of Sanitary and Environmental Engineering.

[45] Arcanjo G.S., Ricci B.C., dos Santos C.R., Costa F.C., Silva U.C., Mounteer A.H., Koch K., da Silva P.R., Santos V.L., Amaral M.C. (2021). Effective removal of pharmaceutical compounds and estrogenic activity by a hybrid anaerobic osmotic membrane bioreactor–membrane distillation system treating municipal sewage. Chem. Eng. J..

[46] GHS (2023). Globally Harmonized System of Classification and Labelling of Chemicals.

[47] Strotmann U., Durand M.-J., Thouand G., Eberlein C., Heipieper H.J., Gartiser S., Pagga U. (2024). Microbiological toxicity tests using standardized ISO/OECD methods—Current state and outlook. Appl. Microbiol. Biotechnol..

[48] Strotmann U., Heipieper H.J., Eberlein C., Mayer P., Birch H., Gartiser S., Pagga U., Daturpalli S., Battagliarin G., McDonough K. (2026). Testing the biodegradability of difficult compounds: A future challenge for the OECD/ISO standardization. Appl. Microbiol. Biotechnol..

